# Exploratory Cross-Cohort Transcriptomic Comparison of Coronary Artery Disease and Non-Obstructive Azoospermia

**DOI:** 10.3390/ijms27177909

**Published:** 2026-09-04

**Authors:** Tengyu Wang, Pengwei Song, Hong Wang, Hongyu Wang, Ruxin Zou, Shuyuan Guo

**Affiliations:** 1 Department of Cardiology, Cardiovascular Institute, The First Affiliated Hospital, Harbin Medical University, 23 Youzheng Street, Harbin 150001, China; wty0710@126.com (T.W.); songpengwei@126.com (P.S.); 815339@hrbmu.edu.cn (H.W.); why6wanghongyu@163.com (H.W.); 2 Department of Radiology, The First Affiliated Hospital, Harbin Medical University, 23 Youzheng Street, Harbin 150001, China; zourx1108@163.com

**Keywords:** coronary artery disease, non-obstructive azoospermia, weighted gene co-expression network analysis, machine learning, single-cell RNA sequencing

## Abstract

Coronary artery disease (CAD) and non-obstructive azoospermia (NOA) have distinct aetiologies. We examined whether separately analysed public transcriptomic cohorts contained overlapping exploratory candidate signals without assuming a shared causal mechanism. We re-analysed five Gene Expression Omnibus datasets using differential-expression screening, weighted gene co-expression network analysis (WGCNA), an archived neural-network candidate ranking, xCell enrichment scoring, and single-cell transcriptomic mapping. Nominal differential-expression screening identified 978 CAD-associated and 2562 NOA-associated candidate transcripts. WGCNA showed a moderate correlation between the MElightyellow module and NOA status (r = 0.58, *p* = 0.007) in GSE45887, a small, imbalanced, non-independent subset of GSE45885. An archived neural-network (NNET) ranking prioritised HSPA1B, PLCL2, ISLR2, STRN, and AQP7 for descriptive analyses; the ranking was generated from the same 20 specimens and is treated as heuristic. xCell produced marker-gene enrichment scores rather than direct measurements of cell abundance or function. Single-cell mapping was descriptive because GSE149512 combined heterogeneous NOA aetiologies with paediatric and adult comparator tissues. The analyses generate hypotheses from separate CAD and NOA cohorts. They do not establish a shared causal pathway, a sex- or age-independent association, temporal sequence, clinical diagnostic utility, or direct correspondence between testicular and coronary cell states.

## 1. Introduction

Non-obstructive azoospermia (NOA), a severe form of male infertility, is defined by the absence of spermatozoa in the ejaculate resulting from impaired spermatogenesis [1,2]. It affects approximately 1% of men and about 10% of infertile men [3]. NOA has heterogeneous aetiologies, including genetic and chromosomal abnormalities, congenital or endocrine disorders, gonadotoxic exposures, systemic disease, and idiopathic forms. Coronary artery disease (CAD) is also heterogeneous and is driven primarily by coronary atherosclerosis and its interaction with established cardiometabolic and inflammatory risk factors [4,5,6].

Population studies have reported associations between impaired male reproductive health and subsequent cardiometabolic morbidity [7,8]. These observations provide epidemiological context but do not show that NOA causes CAD, that CAD causes NOA, or that the two disorders share an initiating mechanism. The disorders have distinct established aetiologies, and the CAD and NOA datasets analysed here were obtained from different participants and biospecimens; they do not constitute a comorbidity cohort.

Metabolic regulation, oxidative-stress responses, inflammation, and extracellular-matrix remodelling are relevant to both reproductive and cardiovascular biology [9,10,11]. However, transcriptomic overlap across peripheral blood and testicular tissue may reflect tissue composition, age, sex, treatment, platform, and other cohort differences. The available data were not designed to test androgen deficiency, sex-hormone mediation, or any cross-organ mechanism directly.

We therefore asked whether independently generated CAD and NOA transcriptomic datasets contained overlapping candidate signals and where those signals were expressed within the available testicular single-cell dataset. This study combines bulk differential-expression screening, WGCNA, an archived candidate-ranking step, marker-gene enrichment scoring, and single-cell mapping. The objective is to generate hypotheses and prioritise candidates for future validation, not to develop a clinical diagnostic model or demonstrate a causal CAD-NOA axis.

## 2. Results

### 2.1. Differential-Expression Screening in CAD and NOA

**Figure 1:**
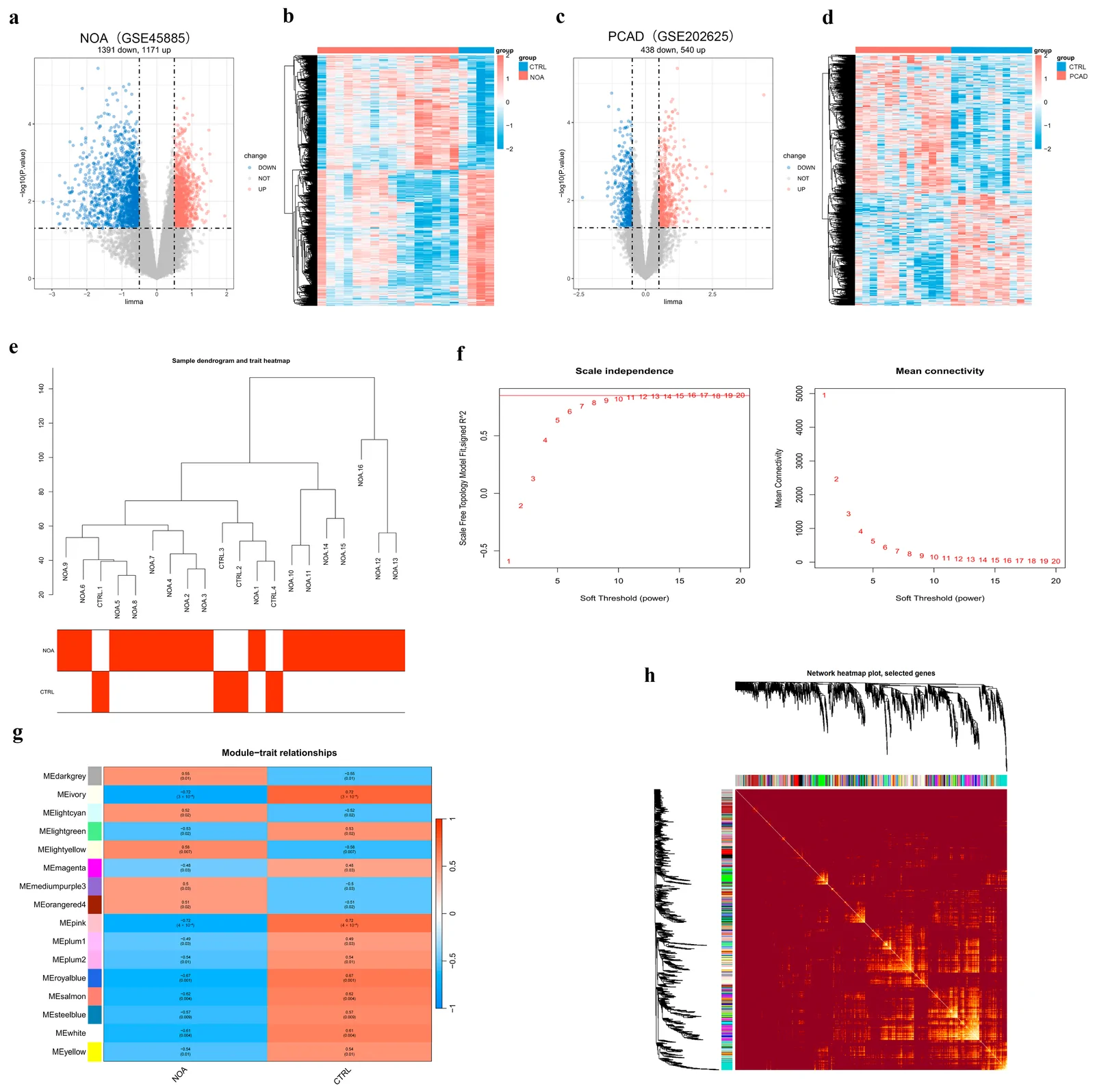
Exploratory differential-expression screening and WGCNA in coronary artery disease and non-obstructive azoospermia. (**a**,**c**) Volcano plots for GSE45885 (NOA) and GSE202625 (CAD). (**b**,**d**) Heatmaps of screened transcripts. (**e**) Sample dendrogram for GSE45887 (16 NOA and 4 controls). (**f**) Soft-threshold diagnostic plots used to select β = 10. (**g**) Module-trait correlations, with MElightyellow correlated with NOA status (r = 0.58, *p* = 0.007). (**h**) Topological-overlap heatmap. GSE45887 is a non-independent subset of GSE45885; its groups are markedly imbalanced, and genome-wide screening used nominal *p* values; all findings are descriptive. The dashed vertical lines in the volcano plot indicate |log2FC| = 1, and the horizontal dashed line indicates *p* = 0.05. Red and blue dots represent significantly upregulated and downregulated genes, respectively, whereas gray dots represent nonsignificant genes. In the WGCNA scale-independence plot, the numbers indicate the candidate soft-thresholding powers, and the horizontal reference line indicates a scale-free topology fit index of R^2^ = 0.85. In the network heatmap, the colored bars indicate different WGCNA co-expression modules, and the heatmap colors represent the degree of topological overlap among genes.

Differential-expression screening was performed separately in the NOA dataset GSE45885 and the CAD dataset GSE202625. Using the prespecified exploratory thresholds of nominal *p* < 0.05 and |log2 fold change| > 1, 2562 NOA-associated candidates (1171 higher and 1391 lower in NOA) and 978 CAD-associated candidates (540 higher and 438 lower in CAD) were retained (Figure 1a–d). Because the screening *p*-values were not adjusted for multiple testing, these transcripts are treated as discovery candidates rather than confirmed differentially expressed genes.

### 2.2. WGCNA Identifies the MElightyellow Candidate Module

WGCNA was applied to GSE45887, which comprised 20 testicular specimens (16 NOA and 4 controls). The sample identifiers correspond to a subset of GSE45885; GSE45887 is therefore non-independent and was not used as validation. Sample clustering identified no outliers requiring exclusion (Figure 1e). The small and markedly imbalanced cohort limits statistical power and the stability of gene–gene correlations and module assignments; the analysis is descriptive and exploratory.

Soft-thresholding powers from 1 to 20 were examined with the pickSoftThreshold function. A power of β = 10 was selected from the scale-free topology fit and mean-connectivity plots (Figure 1f). Meeting this diagnostic criterion does not compensate for the limited sample size.

The MElightyellow module showed the largest positive module-trait correlation with NOA status (r = 0.58, *p* = 0.007) and the corresponding negative correlation with control status (r = −0.58, *p* = 0.007; Figure 1g). Given the 16:4 group imbalance and non-independence from GSE45885, this nominal association was treated as a descriptive result rather than evidence of a stable or reproducible module.

The module eigengene and topological-overlap displays indicated internal co-expression structure (Figure 1h). MElightyellow was carried forward only as a candidate module; its stability, disease specificity, and reproducibility were not established in an independent NOA cohort.

### 2.3. Candidate-Gene Identification and Functional Enrichment

**Figure 2:**
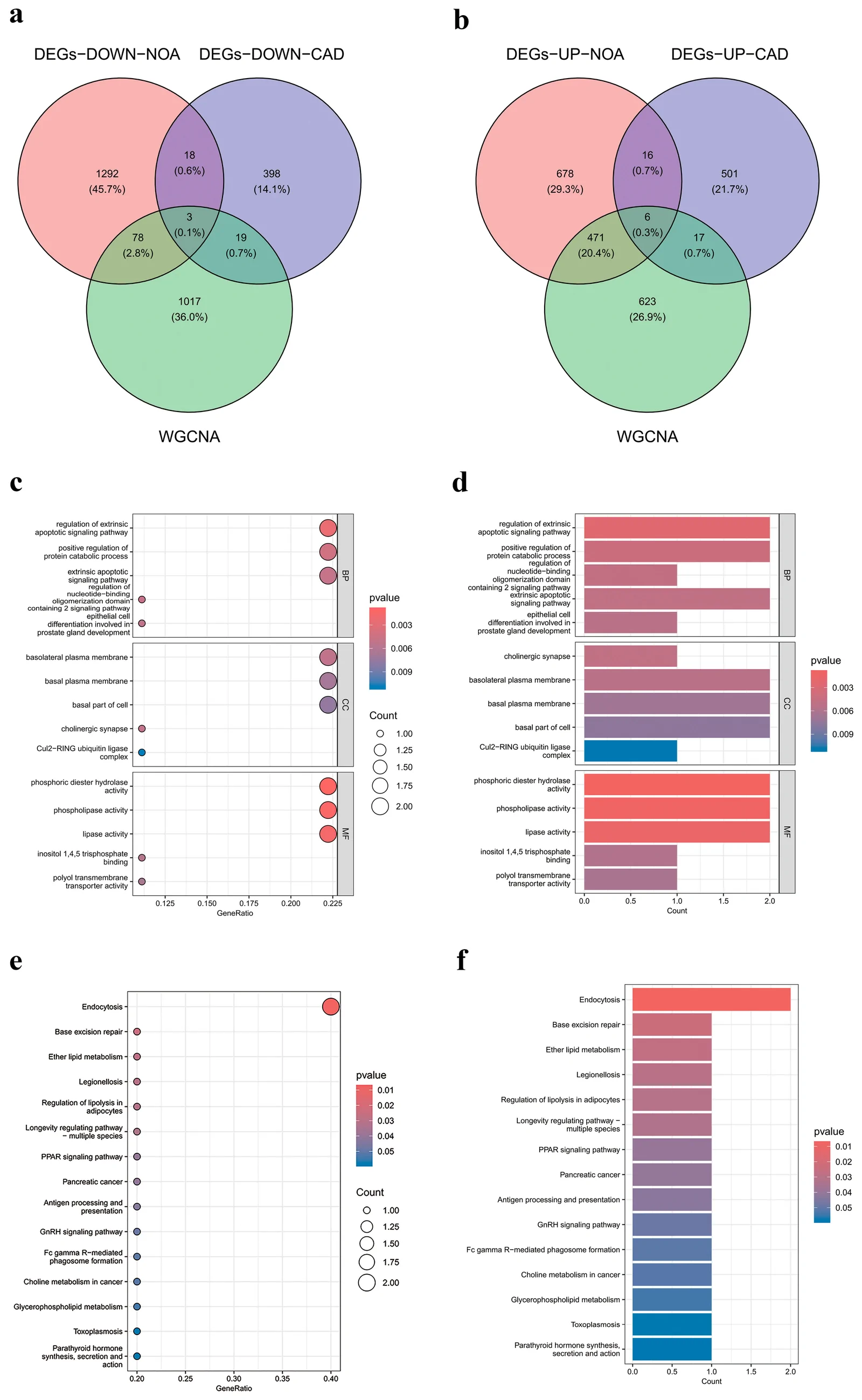
Candidate-gene intersection and exploratory functional annotation. (**a**,**b**) Three-way intersections of CAD screening candidates, NOA screening candidates, and MElightyellow genes. (**c**,**d**) GO annotations. (**e**,**f**) KEGG annotations. Because the input comprised only nine selected genes, the enrichment results are descriptive and do not establish disease, tissue, or cell-type specificity.

The intersection of MElightyellow genes with the nominally screened NOA and CAD transcript sets yielded nine candidate genes (Figure 2a,b). Exploratory Gene Ontology (GO) and Kyoto Encyclopedia of Genes and Genomes (KEGG) analyses linked this small gene set to apoptosis-related signalling, phospholipase activity, inositol 1,4,5-trisphosphate binding, endocytosis, PPAR signalling, GnRH signalling, and glycerophospholipid/ether-lipid metabolism (Figure 2c–f). These annotations represent over-represented terms in the selected gene set; they do not demonstrate that the genes are specific to CAD, NOA, a particular tissue, or a particular cell type.

### 2.4. Candidate Ranking Based on Exploratory NNET

The archived workflow fitted a neural-network model to the nine candidate genes in GSE45887. Because the same 20 specimens contributed to module construction, candidate selection, and model fitting, the workflow could not provide an unbiased estimate of classifier performance. The NNET output was therefore used only to document the heuristic ranking applied to select five genes for downstream descriptive analyses.

**Figure 3:**
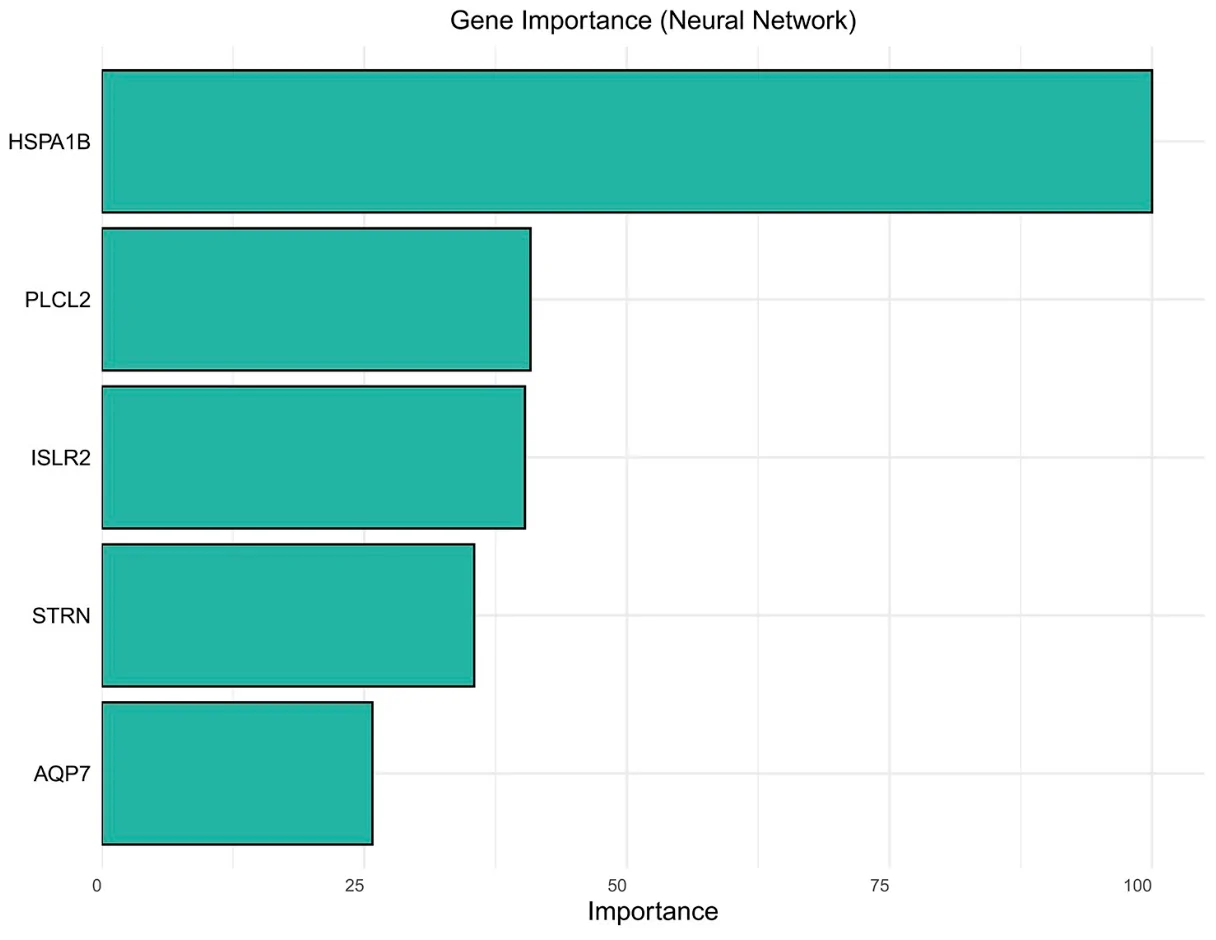
Exploratory NNET-derived ranking used to select five candidates for downstream descriptive analyses. The ranking was generated from the same small GSE45887 dataset used for candidate generation and did not incorporate nested feature selection, alternative importance methods, or independent validation. It documents the heuristic selection of HSPA1B, PLCL2, ISLR2, STRN, and AQP7 and does not represent validated feature importance or diagnostic performance.

The archived NNET ranking identified HSPA1B, PLCL2, ISLR2, STRN, and AQP7 as the five highest-ranked candidates (Figure 3). The workflow did not include SHAP or permutation importance, leave-one-gene-out analysis, hyperparameter sensitivity analysis, or nested feature selection. The five-gene cutoff and ordering are consequently treated as heuristic, and the genes are described as exploratory candidates rather than validated features or biomarkers.

### 2.5. Cross-Dataset Evaluation of Candidate-Gene Expression

Expression of the five heuristically ranked candidates was examined in GSE45887 (NOA versus control) and in a separate CAD dataset, GSE42148 (13 CAD cases aged 40–55 years and 11 controls matched for age, sex, diabetes, and hypertension). The public GSE42148 record does not provide a group mean or standard deviation for age. GSE42148 was used only to assess the direction of gene-expression changes; no diagnostic model was transferred, recalibrated, or evaluated in that cohort. Accordingly, this analysis is a cross-dataset expression check rather than external validation of diagnostic performance.

**Figure 4:**
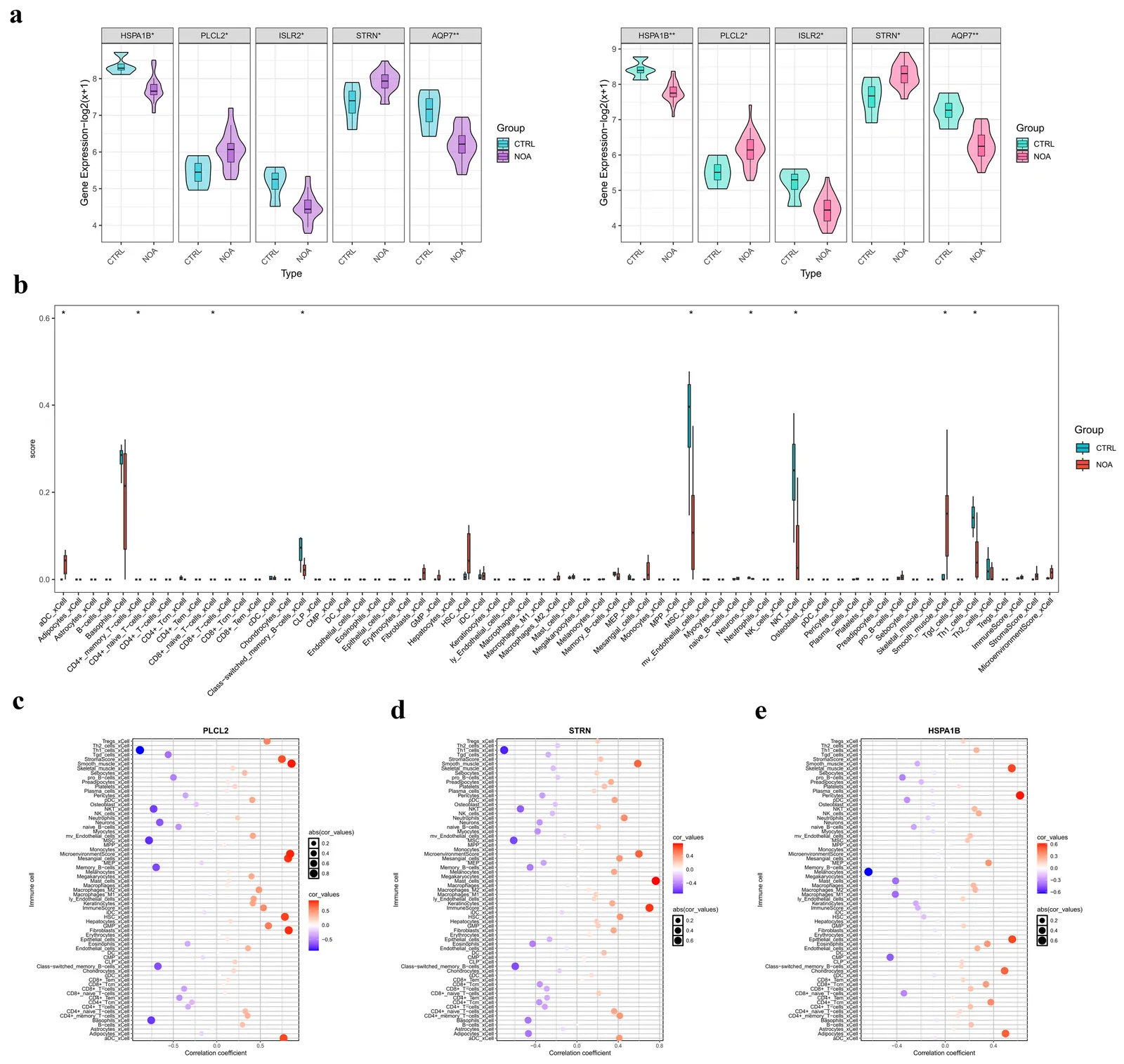
Cross-dataset candidate-gene expression and exploratory xCell scoring. (**a**) Expression of HSPA1B, PLCL2, ISLR2, STRN, and AQP7 in GSE45887 and GSE42148. This panel evaluates expression direction, not classifier performance. (**b**) xCell marker-gene enrichment scores in NOA and control testicular samples. (**c**–**e**) Associations between selected genes and xCell scores. xCell scores are not direct cell counts or measurements of activation or function. * *p* < 0.05, ** *p* < 0.01.

PLCL2 and STRN showed higher expression, whereas HSPA1B, ISLR2, and AQP7 showed lower expression in the displayed comparisons (Figure 4a). These observations support follow-up of the five candidates but do not establish a fixed classification as pathogenic or protective, reversibility, or a shared cellular mechanism across testis and blood.

Because the NOA and CAD measurements came from different tissues, platforms, participants, age distributions, and sex compositions, concordant expression directions cannot be interpreted as direct evidence of the same process in testicular and coronary tissues. No participant-level cross-cohort pooling, age harmonisation, male-only CAD analysis, or coronary-artery single-cell or spatial analysis was performed.

### 2.6. Exploratory xCell Enrichment and Candidate Gene–Cell Signature Associations

xCell was applied to GSE45887 to generate enrichment scores for predefined immune and stromal marker sets. xCell was not trained specifically for testicular tissue, and its scores are influenced by the distinctive cellular composition of the testis. The results are therefore presented as exploratory marker-gene enrichment rather than as evidence of immune infiltration or functional validation.

Several xCell scores differed between the 16 NOA and 4 control specimens (Figure 4b and Figure S1a). The marked group imbalance and lack of a testis-specific reference mean that these differences may reflect cell-composition changes, marker overlap, or technical effects.

Associations between the five candidate genes and xCell scores were summarised using rank correlations (Figure S1b). These associations do not establish regulatory effects on, migration of, or functional changes within the corresponding cell types.

PLCL2 and STRN were positively associated with smooth-muscle-cell-related scores, whereas AQP7 and ISLR2 were negatively associated with those scores in this dataset (Figure 4c,d and Figure S1c,d). The term ‘smooth muscle cell score’ refers to the xCell signature and not to directly measured vascular smooth muscle cells in coronary tissue.

HSPA1B was associated with several xCell scores, including pericyte-, smooth-muscle-cell-, macrophage-, and mast-cell-related signatures (Figure 4e). These score associations do not measure macrophage polarisation, mast-cell degranulation, cell migration, endothelial injury, or other functional states.

Taken together, the xCell results provide marker-set associations that can guide future validation. They do not prove a cellular pathway linking NOA and CAD.

### 2.7. Single-Cell Mapping of Candidate Genes in Testicular Cell Populations

The five candidates were mapped in GSE149512 after application of the study’s quality-control filters. The series includes 7 NOA donors with heterogeneous aetiologies (3 Klinefelter syndrome, 1 AZFa deletion, and 3 idiopathic NOA) and 10 comparator tissues: 5 paediatric or adolescent physiological donors aged 2, 5, 8, 11, and 17 years, and 5 adults with obstructive azoospermia and normal spermatogenesis. Age, developmental stage, comparator indication, and NOA aetiology are therefore inseparable from the pooled group contrast.

**Figure 5:**
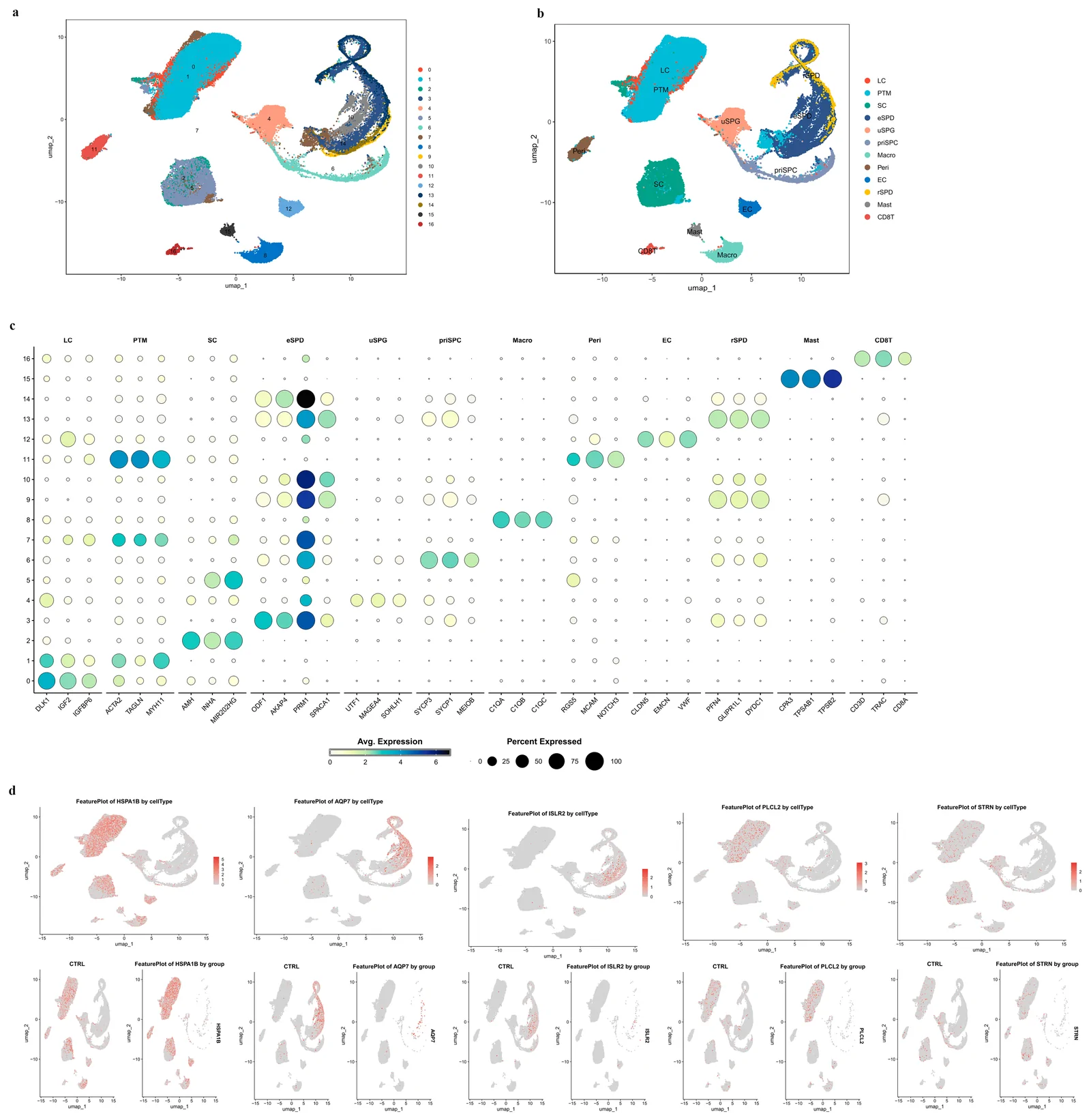
Single-cell mapping of candidate genes in GSE149512. (**a**,**b**) UMAP and t-SNE representations after quality filtering. (**c**) Marker-based annotation of germ-cell and somatic lineages. (**d**) Expression maps for HSPA1B, PLCL2, ISLR2, STRN, and AQP7. The series combines heterogeneous NOA aetiologies, five paediatric or adolescent physiological donors, and five adults with obstructive azoospermia and normal spermatogenesis; pooled group differences are descriptive and not NOA-specific.

Established markers were used to annotate spermatogonia, spermatocytes, early and late spermatids, Sertoli cells, Leydig cells, Peritubular myoid cells (PTMs), endothelial cells, and immune and other interstitial populations (Figure 5a–c). Sertoli and Leydig cells are distinct somatic support populations, not stages of spermatogenesis. Macrophage and mast-cell labels reflect marker expression and do not by themselves establish inflammatory activation or degranulation.

At the donor-group level, the pooled NOA group showed marked depletion of spermatid populations and differences in several somatic and immune-cell proportions (Figure S2a–c). These cross-sectional differences are confounded by age and developmental stage, comparator indication, and NOA aetiology and cannot be attributed specifically to NOA. The cell-proportion plots do not depict a dynamic transition, establish an interaction axis, or show that somatic changes precede germ-cell loss.

HSPA1B, PLCL2, ISLR2, STRN, and AQP7 showed distinct expression patterns across annotated testicular cell populations and study groups (Figure 5d and Figure S2d–g). These patterns provide descriptive localisation within this heterogeneous dataset but do not define NOA-specific effects, demonstrate that any gene initiates NOA, or link testicular cells directly to coronary vascular cells.

### 2.8. CellChat Inference and Pseudotime Ordering of PTM-Related Cell States

**Figure 6:**
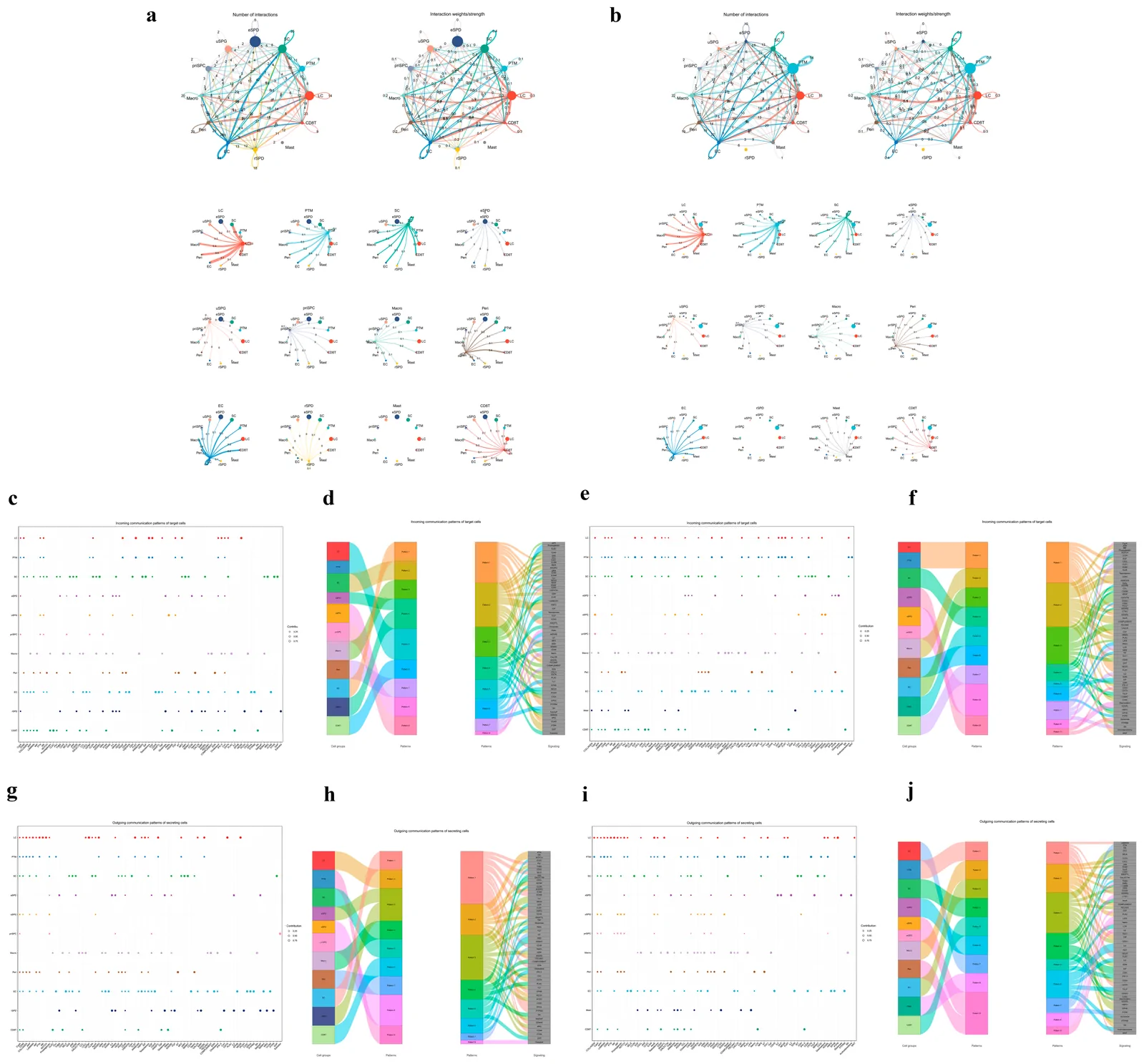
CellChat-inferred communication patterns in the GSE149512 comparator and NOA groups. (**a**,**b**) Inferred ligand-receptor networks. (**c**–**j**) Outgoing and incoming communication-pattern summaries. The pooled comparison is confounded by age, developmental stage, comparator indication, and NOA aetiology; these computational estimates are descriptive and require independent protein-level and functional validation. In subfigures (**a**,**b**), different colors represent different cell populations, and the same color scheme is used consistently across all communication networks. Directed edges indicate the direction of cell-cell communication from sender to receiver populations. The numerical labels indicate the number of inferred interactions between the corresponding cell populations. In the “Number of interactions” networks, edge width is proportional to the number of inferred interactions, whereas in the “Interaction weights/strength” networks, edge width represents the overall communication strength. Edge colors correspond to the source cell populations.

CellChat was used to infer ligand-receptor communication patterns from the testicular single-cell expression data (Figure 6). Differences in inferred androgen-related and complement-related networks were observed between the annotated groups. CellChat estimates communication probabilities from gene-expression data and a prior interaction database; it does not directly measure circulating hormones, receptor activation, complement activity, or physical cell–cell interactions.

**Figure 7:**
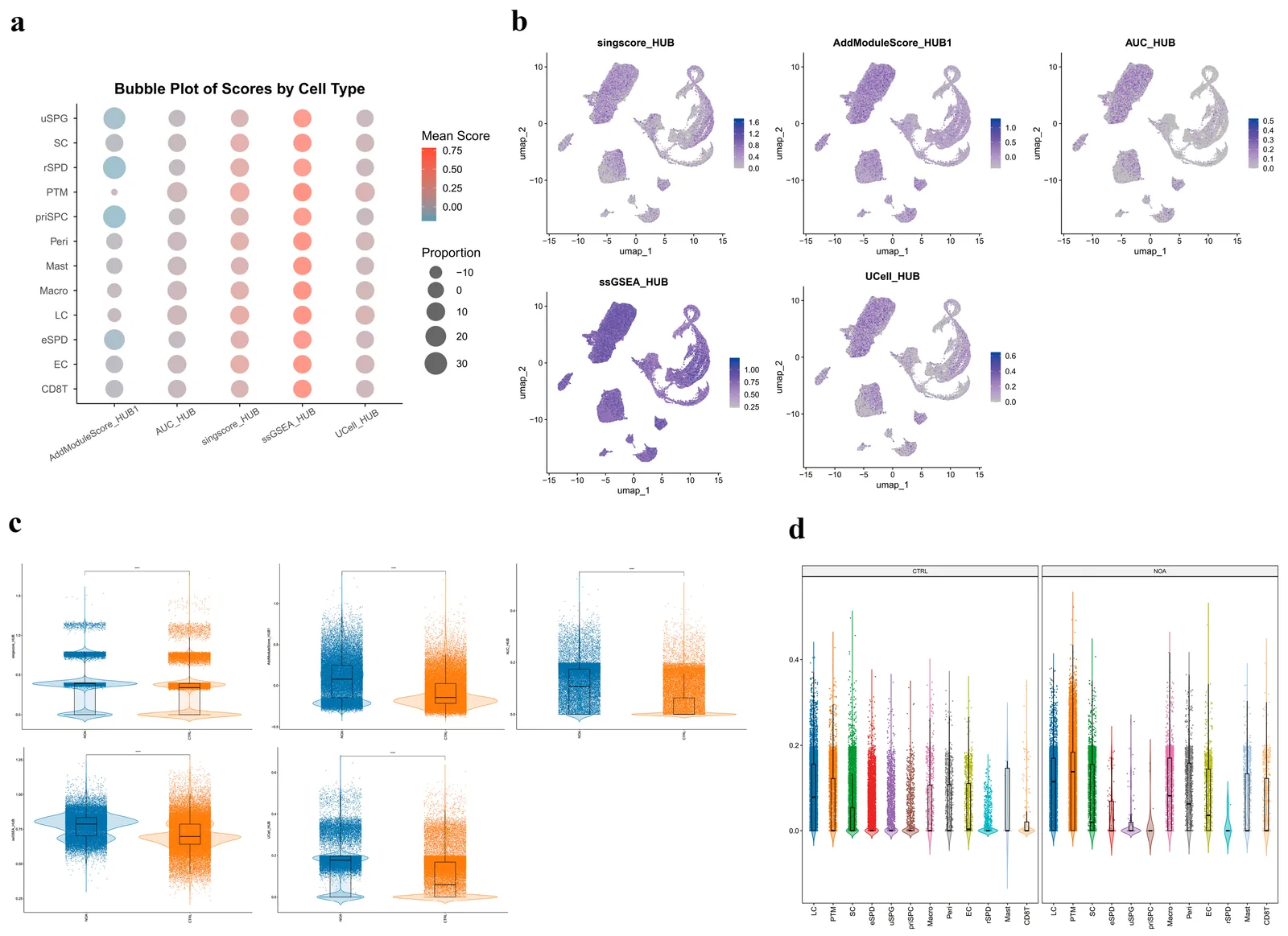
Candidate-gene-set scores across annotated testicular cell populations. (**a**) Aggregated score summaries. (**b**) UMAP score maps. (**c**,**d**) Score distributions by study group and cell type. All methods use the same heterogeneous dataset; group differences are confounded by age, developmental stage, comparator indication, and NOA aetiology, and concordance is not independent validation. **** *p* ≤ 0.0001.

Five gene-set scoring methods (singscore, AddModuleScore, AUCell, ssGSEA, and UCell) were applied to the same single-cell dataset. PTMs had relatively high scores across several visualisations (Figure 7). Concordance among algorithms applied to the same dataset does not constitute independent biological replication and does not establish PTMs as a causal nexus.

**Figure 8:**
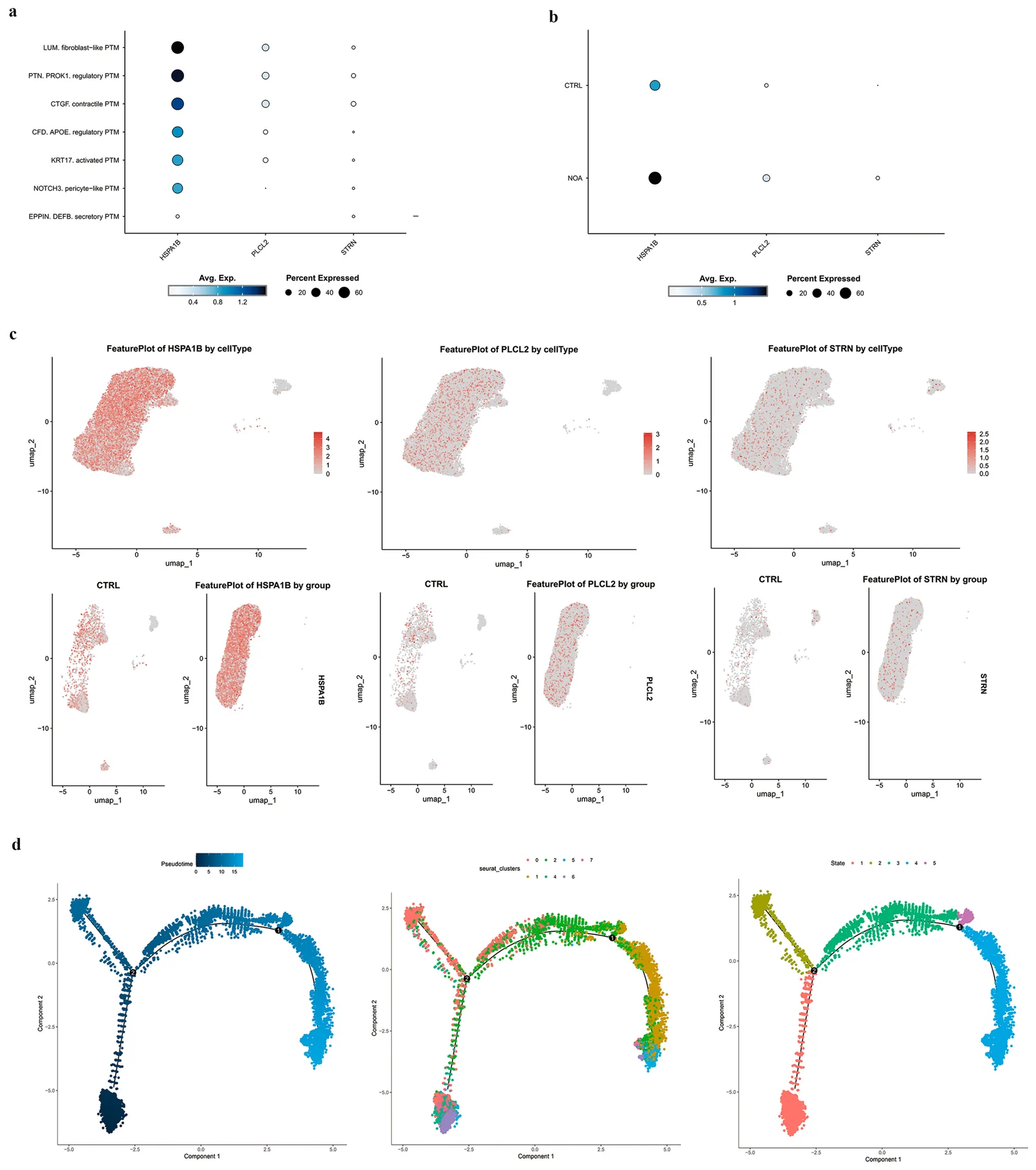
PTM-related subclusters and Monocle2 pseudotime ordering in GSE149512. (**a**,**b**) Candidate-gene expression by subcluster and study group. (**c**) Expression maps. (**d**) Inferred pseudotime trajectory with five states. The underlying donor groups are heterogeneous and age-confounded; pseudotime is a computational ordering of cross-sectional cell states and does not establish temporal sequence or causality. Cells were ordered along the inferred pseudotime trajectory, and the numerical labels indicate the branch points identified in the reconstructed trajectory.

Subclustering of PTM-associated cells yielded seven retained subclusters after exclusion of two low-quality clusters (Figure 8a–c). HSPA1B, PLCL2, and STRN showed distinct expression distributions among the annotated PTM-related states and study groups. The labels ‘contractile’, ‘pericyte-like’, ‘fibroblast-like’, and ‘regulatory’ are transcriptome-based annotations and do not demonstrate phenotypic conversion or functional equivalence to coronary vascular cells.

Monocle2 was used to order PTM-related cells along a data-driven pseudotime trajectory with five states (Figure 8d and Figure S3b,c). The selected root and inferred branch structure represent an organisation of transcriptionally similar cell states. This ordering does not represent a chronological lineage, longitudinal disease course, or observed cell differentiation.

HSPA1B, AQP7, and ISLR2 tended to show higher expression in one region of the inferred ordering, whereas PLCL2 and STRN tended to show higher expression in other regions. These pseudotime-associated patterns generate a testable hypothesis about PTM-related states. They do not establish that HSPA1B loss occurs first, that PLCL2 or STRN is activated subsequently, or that any of these genes regulates stromal remodelling.

## 3. Discussion

### 3.1. Interpretation of Cross-Dataset Transcriptomic Overlap

This study identified candidate transcripts that appeared in separate analyses of CAD blood and NOA testicular samples. No patient-level expression matrices were pooled across diseases. In GSE202625, the CAD and no-plaque groups were similar in sex distribution (21/27 and 20/25 male, respectively) and age (63 ± 8 and 62 ± 7 years, respectively), reducing within-dataset confounding for the CAD contrast. Nevertheless, the CAD series included women; no sex-stratified analysis was performed. GSE42148 reported only an age range of 40–55 years, and participant-level ages were unavailable for the NOA microarray cohorts. The cross-cohort overlap therefore cannot support a male-specific, androgen-mediated, age-independent, or premature-CAD mechanism, nor show that one disease precedes or causes the other.

The enrichment results highlight pathways relevant to metabolism, endocrine signalling, stress responses, and extracellular-matrix biology. Such pathway-level convergence may motivate follow-up studies, but it does not demonstrate synergy, antagonism, or a single shared upstream programme. Given the intersection of only nine candidates and the use of nominal genome-wide screening, false-positive selection remains possible.

PTMs and vascular smooth muscle cells arise from different developmental contexts [12,13]. They may share contractile markers and some stress-responsive programmes, but this represents a functional analogy rather than an ontogenetic continuum or molecular homology [14,15]. Because no coronary-artery single-cell data were analysed, the present study cannot directly compare PTMs with coronary vascular smooth muscle cells.

### 3.2. HSPA1B and the Other Heuristically Ranked Genes as Exploratory Candidates

HSPA1B was highly ranked in the archived NNET output and showed variable expression across testicular cell states. HSPA1B encodes an inducible HSP70-family chaperone broadly involved in proteostasis and cellular stress responses [16,17,18,19]. These known functions provide biological context but do not establish HSPA1B as an upstream regulator in CAD or NOA.

The frequently cited mouse HSP70-2 knockout infertility phenotype concerns HSPA2 rather than HSPA1B [20] and therefore cannot be used as direct validation of the present HSPA1B association. Likewise, lower HSPA1B expression in cross-sectional data cannot be described as transcriptional collapse, a protective brake, or a permissive switch without perturbation experiments.

PLCL2, ISLR2, STRN, and AQP7 also require gene-specific validation. The five-gene set was selected by an archived model-specific ranking without nested feature selection, alternative importance methods, gene-removal sensitivity analysis, or hyperparameter stability testing. Its composition and ordering are heuristic and may differ in other datasets.

### 3.3. Testicular Cell Context and the Limits of Cross-Organ Analogy

The most direct single-cell observation in NOA was the depletion or arrest of germ-cell populations. Sertoli cells, Leydig cells, PTMs, vascular cells, and immune cells provide important somatic context, but their cross-sectional transcriptomic differences cannot be assumed to precede or cause germ-cell failure.

CellChat, xCell, and gene-set scoring provide computational summaries of expression patterns. They do not directly measure androgen signalling, complement activation, immune-cell function, extracellular-matrix deposition, blood–testis-barrier integrity, or cell migration. The present results cannot establish these unmeasured processes.

Similarly, gene expression in CAD blood samples cannot be projected onto coronary smooth muscle cells or plaques. The present analysis therefore does not claim direct cross-organ cellular similarity.

The present findings are therefore framed as associations among independently observed transcriptomic signals, not as a stromal-immune circuit that drives both diseases.

### 3.4. Translational Implications and Validation Priorities

No candidate from this study is ready for clinical diagnosis, cardiovascular screening, or therapeutic targeting. The five genes remain research candidates that require evaluation in larger, balanced cohorts with harmonised covariates, independent validation, adult testicular and coronary-tissue confirmation, and functional experiments.

### 3.5. Limitations

This study has substantial limitations. First, genome-wide screening used nominal *p* < 0.05 with an effect-size threshold and no study-wide multiplicity correction, increasing the risk of false positives. Second, the NOA datasets were small and markedly imbalanced (GSE45885, 27 versus 4; GSE45887, 16 versus 4). GSE45887 contains the same 4 control specimens and 16 NOA specimens that are also present in GSE45885; it is a non-independent subset, not a validation cohort. The 16:4 analysis has limited statistical power and cannot establish module stability or reproducibility. Third, WGCNA and the archived NNET ranking used these same 20 specimens. No nested feature selection, independent model validation, alternative feature-importance analysis, leave-one-gene-out analysis, or hyperparameter sensitivity analysis was conducted; the archived NNET output is only a heuristic record of candidate selection. Fourth, GSE202625 included women, although its CAD and no-plaque groups were similar in sex and age distributions; no male-only or sex-stratified analysis was performed. The public GSE42148 record provides an age range, but no mean ± standard deviation, and participant-level ages for the NOA microarray cohorts were unavailable. No cross-cohort age harmonisation was possible. Fifth, BMI, lipid profile, smoking, CAD extent, disease duration, hormone measurements, and other clinical covariates were not uniformly available and were not adjusted across series. Sixth, GSE45885 includes several histopathological patterns, and GSE149512 combines three distinct NOA aetiologies with five paediatric or adolescent physiological donors and five adults with obstructive azoospermia and normal spermatogenesis; pooled single-cell differences are therefore not NOA-specific. Seventh, xCell is not testis-specific, and xCell, CellChat, gene-set scoring, and pseudotime are computational inferences rather than direct measurements. Finally, no independent external cohort or functional experiment was included; the cross-tissue design and absence of coronary-tissue single-cell data preclude causal or clinical conclusions.

## 4. Materials and Methods

### 4.1. GEO Data Collection and Preliminary Processing

This study re-analysed five publicly available Gene Expression Omnibus (GEO) series [21,22,23,24,25]. The 144 accession-level specimens (90 disease-labelled and 54 comparator-labelled) were summed across series and do not represent a jointly phenotyped CAD-NOA cohort or 144 unique participants. Direct comparison of the public sample identifiers shows that all 16 NOA specimens and all 4 control specimens in GSE45887 are also present in GSE45885. GSE45887 is therefore a non-independent subset of GSE45885 and was not treated as an independent validation cohort [22,23].

GSE202625 comprised whole-blood RNA sequencing from 27 participants with severe CCTA-defined CAD (at least 70% stenosis in one vessel) and 25 participants without detectable coronary plaque [21]. The CAD and no-plaque groups were similar in age (63 ± 8 versus 62 ± 7 years) and sex distribution (21/27 versus 20/25 male), although hypertension differed between groups in the source report. GSE42148 comprised peripheral-blood microarrays from 13 angiographically confirmed CAD cases aged 40–55 years and 11 asymptomatic controls with normal electrocardiograms matched for age, sex, diabetes, and hypertension; a group mean and standard deviation for age were not deposited [20]. GSE45885 included 27 NOA accession-level biopsy specimens labelled as postmeiotic arrest (*n* = 11), meiotic arrest (*n* = 7), premeiotic arrest (*n* = 2), or Sertoli-cell-only syndrome (*n* = 7), with 4 normal-spermatogenesis controls [22]. GSE45887 comprised a non-independent subset of 16 NOA and the same 4 control specimens [23]. GSE149512 contained testicular single-cell data from 7 NOA donors (3 Klinefelter syndrome, 1 AZFa deletion, and 3 idiopathic NOA), 5 paediatric or adolescent physiological donors, and 5 adults with obstructive azoospermia and normal spermatogenesis [24].

The public records and source reports were used to summarise the available cohort definitions in Table 1 [21,22,23,24,25]. For the CAD series, available selection variables included CCTA-defined severe CAD or absence of plaque in GSE202625 and angiographically confirmed CAD or asymptomatic normal-electrocardiogram control status in GSE42148. For the NOA microarray series, groups were defined by testicular histopathology and normal-spermatogenesis comparators. The single-cell series included heterogeneous genetic and idiopathic NOA aetiologies and age- and indication-heterogeneous comparator tissues. Participant-level NOA age, complete hormone profiles, CAD vessel counts, BMI, lipid profiles, smoking, disease duration, treatment, and other clinical covariates were not uniformly deposited. These variables could not be harmonised or adjusted in the re-analysis.

Processed expression matrices were analysed separately within each series; no cross-platform participant-level matrix was constructed. For array datasets, probe identifiers were mapped to gene symbols using platform annotations, probes without a mapped symbol were excluded, and expression values from multiple probes mapping to the same symbol were averaged.

### 4.2. Differential-Expression Screening

Differential-expression screening was performed separately within GSE202625 and GSE45885 using the empirical-Bayes variance-moderation framework in limma (version 3.54.2) [26]. Candidate transcripts were defined by nominal *p* < 0.05 and |log2(fold change)| > 1. No genome-wide false-discovery-rate correction was applied at this screening stage; the retained transcripts are therefore exploratory candidates.

### 4.3. Weighted Gene Co-Expression Network Analysis (WGCNA)

WGCNA was performed in GSE45887 with the WGCNA R package [27]. Pairwise correlations, adjacency, topological overlap, hierarchical clustering, and module-trait correlations were calculated using the 20 available specimens (16 NOA and 4 controls). These specimens form a non-independent subset of GSE45885. Soft-thresholding powers from 1 to 20 were examined, and β = 10 was selected from the scale-free topology and mean-connectivity diagnostics. Given the small 16:4 design, limited statistical power, and lack of an independent cohort, module stability and reproducibility could not be established.

### 4.4. Cross-Disease Candidate-Gene Intersection

A three-way intersection of the MElightyellow genes, nominal NOA screening candidates, and nominal CAD screening candidates was generated with the VennDiagram R package. The resulting nine genes were carried forward as an exploratory candidate set; this intersection did not confer disease or tissue specificity.

### 4.5. Functional Enrichment Analysis

GO and KEGG annotations were generated with clusterProfiler (version 4.14.6). When clusterProfiler returned adjusted *p* values, the Benjamini–Hochberg method was used. Because the enrichment analysis included only nine selected genes, it was treated as descriptive and not as independent pathway validation.

### 4.6. Exploratory NNET-Based Candidate Ranking

The archived workflow fitted an NNET model to the nine candidate genes in GSE45887. The same 20 specimens were used for WGCNA-based candidate generation and model fitting; therefore, classifier performance could not be estimated independently of the feature-selection process.

The archived workflow did not implement nested feature selection or independent validation, and documentation of regularisation, early stopping, and hyperparameter stability was not available. The NNET output was therefore used only to document the heuristic candidate ordering used in downstream descriptive analyses.

The fitted NNET produced a model-specific variable-importance ranking. No additional model-interpretation or robustness analyses, such as SHAP or permutation importance, leave-one-feature-out sensitivity analysis, or hyperparameter sensitivity analysis, were performed. The five highest-ranked genes were retained heuristically for downstream expression mapping and are described as exploratory candidates, not validated biomarkers.

### 4.7. Exploratory xCell Marker-Gene Enrichment Analysis

xCell was used to calculate enrichment scores for predefined immune and stromal marker sets [28]. Group comparisons used Wilcoxon rank-sum tests, and gene-score associations used Spearman correlations. xCell scores are not direct cell proportions or measures of activation, migration, polarisation, or degranulation. Because xCell is not testis-specific, the testicular-tissue analyses were considered exploratory.

### 4.8. Single-Cell RNA-Sequencing Data Processing and Cell-Type Annotation

Single-cell mapping used GSE149512 [28]. The series contains 7 NOA donors with heterogeneous aetiologies, 5 paediatric or adolescent physiological donors, and 5 adults with obstructive azoospermia and normal spermatogenesis. A Seurat object was generated after applying the stated quality-control thresholds. Because age, developmental stage, comparator indication, and NOA aetiology were confounded with study group, pooled contrasts were used only for descriptive localisation and not as NOA-specific estimates.

Normalisation, variable-feature selection, scaling, principal-component analysis, neighbour-graph construction, clustering, and UMAP visualisation were performed with Seurat. Cell types were annotated using established markers and reference databases.

Cell-type proportions and within-cell-type expression differences were summarised descriptively. No age-stratified, adult-only, or aetiology-specific analysis was performed. Cell-level *p* values were not treated as substitutes for donor-level replication, and no claim of NOA specificity or functional activation was based solely on marker expression.

### 4.9. CellChat-Based Communication Analysis

CellChat (version 1.6.1) was used to infer ligand-receptor communication probabilities from normalised expression and the CellChatDB interaction prior. The outputs represent computationally inferred communication patterns and do not directly measure hormone concentrations, protein activation, complement activity, or physical interactions.

### 4.10. PTM Subclustering and Pseudotime Analysis

PTM-related cells were subclustered using expression-based annotations. Monocle2 (version 2.36.1) was used to order cells along a pseudotime trajectory [29]. Pseudotime was interpreted as a data-driven ordering of transcriptionally related cross-sectional cell states, not as evidence of chronological disease progression, lineage tracing, or a causal sequence.

### 4.11. Statistical Analysis

Analyses and figures were generated in R (version 4.2.3). Unless specifically identified as adjusted, *p* values were nominal, and *p* < 0.05 was used for exploratory screening. No study-wide multiplicity correction or harmonised participant-level covariate adjustment was performed.

## 5. Conclusions

This exploratory study reports candidate transcripts detected in separate CAD and NOA datasets and describes their expression in one heterogeneous testicular single-cell series. The findings are constrained by non-independent and imbalanced NOA microarray data, mixed-sex and age-different CAD cohorts, unavailable clinical covariates, and age-, indication-, and aetiology-confounded single-cell groups. They do not establish a shared CAD-NOA mechanism, an androgen-mediated association, temporal sequence, diagnostic performance, or therapeutic utility. HSPA1B, PLCL2, ISLR2, STRN, and AQP7 remain unvalidated candidates.

## Figures and Tables

**Table 1:** Characteristics of the NCBI GEO datasets.

Analytic Role	Accession ID	Cohort Definition	Platform	Biospecimen	Disease (*n*)	Comparator (*n*)
CAD screening	GSE202625	CCTA-defined CAD; age 63 ± 8 years; 21/27 male	RNA-seq	Whole blood	27	25 without plaque; age 62 ± 7 years; 20/25 male
NOA screening	GSE45885	NOA: 11 postmeiotic, 7 meiotic, 2 premeiotic arrest, 7 SCOS	Microarray	Testicular biopsy	27	4 with normal spermatogenesis
WGCNA/NNET	GSE45887	Non-independent GSE45885 subset: 7 postmeiotic, 4 meiotic arrest, 5 SCOS	Microarray	Testicular biopsy	16	4 with normal spermatogenesis
CAD expression	GSE42148	CAD; age 40–55 years; mean ± SD not deposited	Microarray	Peripheral blood	13	11 controls matched for age, sex, diabetes, and hypertension
Single-cell mapping	GSE149512	3 Klinefelter syndrome, 1 AZFa deletion, 3 idiopathic NOA	scRNA-seq	Testis	7	5 paediatric physiological donors; 5 adults with OA and normal spermatogenesis

Counts are accession-level specimens, not unique participants. All GSE45887 specimens are present in GSE45885; GSE45887 is a non-independent subset and not a validation cohort. GSE202625 included both sexes, with similar sex and age distributions in the CAD and no-plaque groups. GSE42148 reported only an age range. GSE149512 included heterogeneous NOA aetiologies, five paediatric or adolescent physiological donors, and five adults with obstructive azoospermia and normal spermatogenesis. OA, obstructive azoospermia; SCOS, Sertoli-cell-only syndrome.

## Data Availability

The original data presented in the study are openly available from the Gene Expression Omnibus and are available at https://www.ncbi.nlm.nih.gov/geo/ (accessed on 20 August 2026) with the permission of Gene Expression Omnibus under accessions GSE202625, GSE45885, GSE45887, GSE42148, and GSE149512.

## Supplementary Materials

The following supporting information can be downloaded at https://www.mdpi.com/article/10.3390/ijms27177909/s1.

## Abbreviations

CAD	Coronary artery disease
NOA	Non-obstructive azoospermia
PTM	Peritubular myoid cell
DEG	Differentially expressed gene
WGCNA	Weighted gene co-expression network analysis
NNET	Neural network
GEO	Gene Expression Omnibus
GO	Gene Ontology
KEGG	Kyoto Encyclopedia of Genes and Genomes
