# Supplementary material for: Exploratory Cross-Cohort Transcriptomic Comparison of Coronary Artery Disease and Non-Obstructive Azoospermia
Source: Int J Mol Sci. 2026 Sep 4;27(17):7909. doi: 10.3390/ijms27177909 (PMC13566673; doi:10.3390/ijms27177909)
Supplement: Supplementary file 1 [file ijms-27-07909-s001.zip › ijms-4454301-supplementary.pdf]

Supplementary Materials

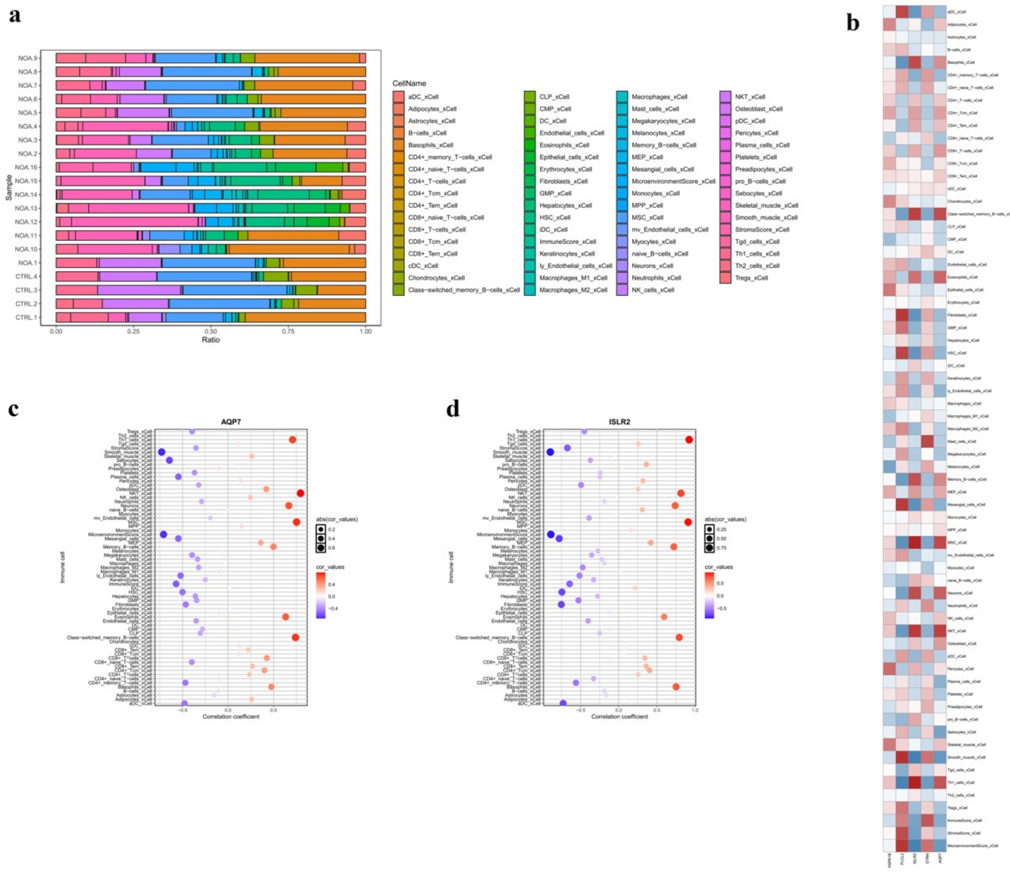

Figure S1. Immune infiltration analysis reveals a dichotomous immunomodulatory landscape bridging NOA and PCAD. (a) Stacked bar charts of the single-sample proportions of each cell type in individual samples, confirming that there is a similar change pattern in the compositional of the disease group. (b) Pearson correlation heat map between the five core feature genes and the xCell-estimated microenvironmental infiltration abundances. (c, d) The correlations between core feature genes - AQP7, ISLR2 and particular immune cell subpopulations are presented through correlation bubble plots.

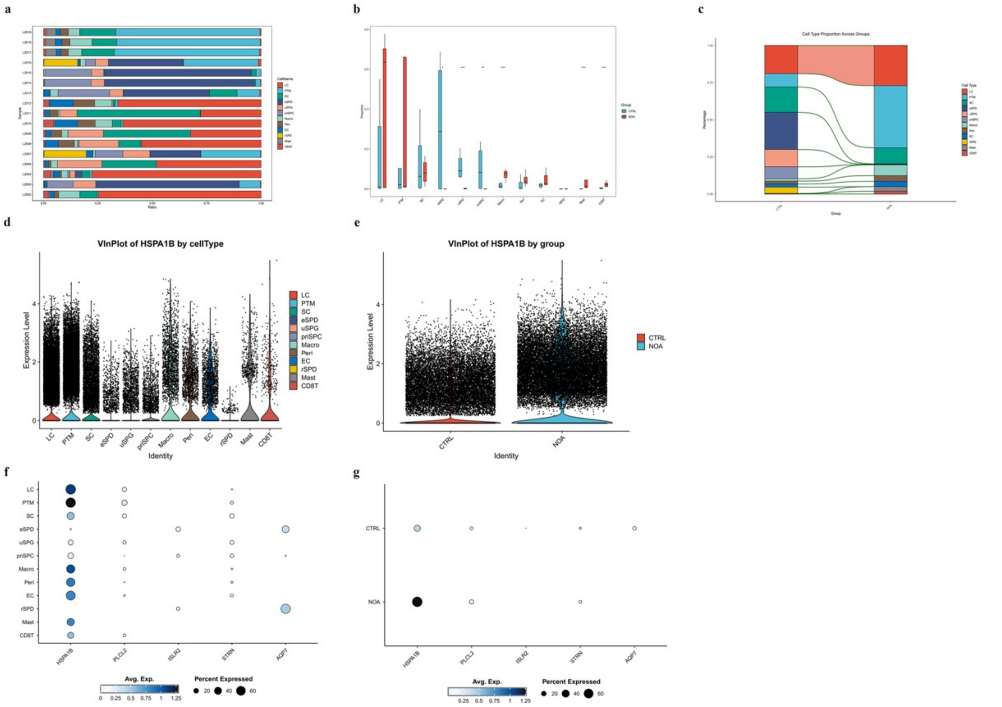

Figure S2. Single-cell transcriptomic profiling of testicular cell lineages and in situ validation of the five-biomarker diagnostic panel in non-obstructive azoospermia. (a, b, c) Analysis of single-cell subpopulation abundance by stacked group bar plots and bar charts, which show the relative proportions and compositional differences of different cells between the normal control (CTRL) and disease groups. (d, e, f, g) Expression analysis in situ through violin plots and feature plots indicating the cell-type specificity of localisation and disease-related expression alterations of the core biomarkers.

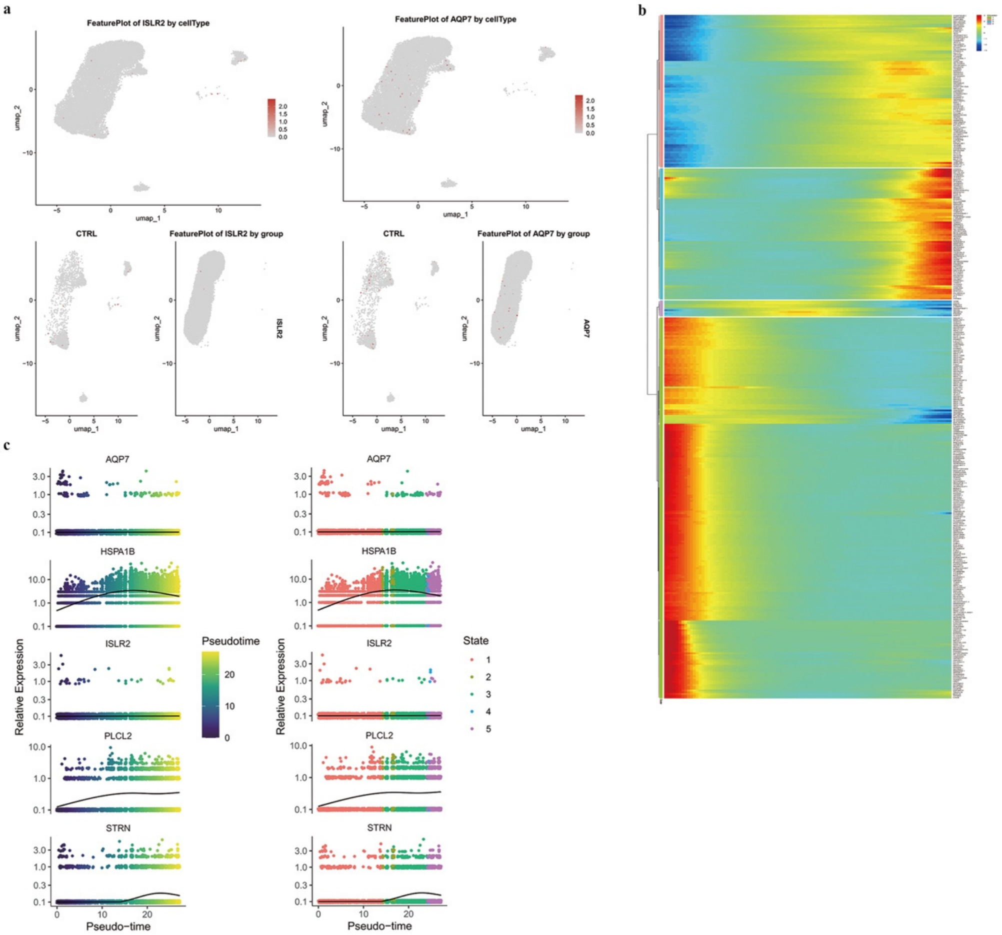

Figure S3. The subcluster analysis of the peritubular myoid cells demonstrates polarized expression dynamics and pseudotime trajectory of the core diagnostic panel in NOA. (a) UMAP feature plots at high resolution of the core biomarkers (ISLR2 and AQP7) across the global testicular landscape and the isolated stromal embedding space. (b) Time-based heatmap which depicts clustered expression pattern of co-expressed genes on the pseudotime continuum and which clusters into coordinated gene modules corresponding to the phenotypic transition. (c) Kinetic expression curves that plot the relative abundance of the five core biomarkers (HSPA1B, PLCL2, ISLR2, STRN, and AQP7) on the pseudotime axis.
